# Real-World Utility of the Host-Response MeMed BV Test in a Pediatric Emergency Department: A Non-Randomized Study with Optimized Antimicrobial and Diagnostic Stewardship

**DOI:** 10.3390/children12091129

**Published:** 2025-08-27

**Authors:** Panagiota Diamantopoulou, Sofia Karagiannidou, Chrysanthi-Eleni Loizou, Vassiliki Papaevangelou, Garyfallia Syridou

**Affiliations:** Third Department of Pediatrics, University General Hospital ATTIKON, School of Medicine, National and Kapodistrian University of Athens, 12462 Athens, Greece; padiama@med.uoa.gr (P.D.); sofikar@med.uoa.gr (S.K.); chrysalenal@med.uoa.gr (C.-E.L.); vpapaev@med.uoa.gr (V.P.)

**Keywords:** rapid host protein test, pediatric emergency department, antibiotic stewardship, viral, bacterial

## Abstract

**Background**: Differentiating between bacterial and viral infections in pediatric emergency care is challenging, often leading to unnecessary antibiotic use. The MeMed BV (MMBV) test is a host-response assay designed to differentiate bacterial from viral infections, but real-world data in pediatric settings remain limited. **Methods**: We conducted a pragmatic, single-center, prospective cohort study to assess the clinical utility of MMBV in children with acute respiratory infections or fever without source. Patients were assigned to standard of care (SOC) or MMBV testing (SOC+MMBV) based on time of presentation to the emergency department. The primary outcome was antibiotic prescribing. Secondary outcomes included diagnostic test utilization, hospitalization rates, and length of stay. Analyses were stratified by hospitalization status, clinical severity [National Institute for Health and Care Excellence (NICE) traffic light system], and patient age. **Results**: From July 2023 to April 2024, 343 patients were enrolled (171 SOC, 172 SOC+MMBV). In the SOC+MMBV arm, reduced antibiotic prescribing was observed among outpatients and those with non-severe signs and symptoms. Antibiotic prescribing was significantly reduced in children under five years with a low-risk profile, according to the NICE traffic light system (26.3% vs. 7.5%; *p* = 0.034). Multiplex PCR testing was significantly reduced in the SOC+MMBV group (28.7% vs. 16.3%; *p* = 0.006) compared to SOC for both inpatients and outpatients. No significant differences were observed in overall diagnostic test use or length of stay. **Conclusions**: MMBV improved antibiotic and diagnostic stewardship in a real-world pediatric ED setting, significantly reducing unnecessary antibiotic use among low-risk children under five and minimizing unnecessary multiplex PCR testing across the cohort.

## 1. Introduction

Accurately distinguishing between bacterial and viral infections in febrile pediatric patients presenting to the emergency department (ED) is a persistent challenge, particularly when symptoms are nonspecific [1]. This diagnostic uncertainty often leads to unnecessary antibiotic prescribing, increasing the risk of adverse events and contributing to antimicrobial resistance (AMR) [2,3,4]. In Greece, AMR is a significant public health threat, with a high prevalence of multidrug-resistant (MDR) and extensively drug-resistant (XDR) pathogens such as *Klebsiella pneumoniae*, *Acinetobacter baumannii*, and *Pseudomonas aeruginosa* [5]. Despite national antimicrobial stewardship initiatives, Greece continues to report the second-highest antibiotic consumption in the EU/EEA [6].

Children, especially those under five years, are particularly vulnerable to inappropriate antibiotic exposure, which may disrupt the developing microbiome and increase the risk of antimicrobial resistance and allergic diseases [7,8,9,10]. Today, pediatric populations remain underrepresented in national surveillance efforts, perpetuating gaps in optimal antibiotic use guidance [11]. Enhancing diagnostic accuracy in pediatric tertiary care centers, where patients often present with complex comorbidities, is, therefore, critical to improving antimicrobial stewardship. Notably, new tools must exhibit both high sensitivity and specificity, as reducing unnecessary antibiotic prescriptions to children with likely viral infections cannot come at the cost of missed bacterial infections.

Diagnostic tools such as C-reactive protein (CRP) and procalcitonin (PCT) are commonly used to support clinical decision-making despite the ongoing debate on their sensitivity for potentially serious bacterial infections and utility in changing antibiotic prescribing [12,13,14,15]. Elevated CRP may occur in viral illnesses and inflammatory conditions, while PCT has not consistently reduced antibiotic prescribing in pediatric populations [16]. Regarding PCT use in the ED, it seems that without standardized clinical protocols, PCT results just above the threshold could lead to unnecessary antibiotic prescription, as well as overuse of diagnostic tests [17,18]. Pathogen-based testing, including multiplex PCR panels, identifies causative organisms but has shown limited impact on antibiotic prescribing [19,20].

To address the limitations of individual biomarkers, advanced host-response diagnostics have emerged, combining multiple biomarkers to provide a more integrated insight into the immune response and improve infection differentiation [21]. The MeMed BV (MMBV) test (MeMed, Andover, MA, USA) is an advanced regulatory-cleared host-response assay, measuring Tumor Necrosis Factor-Related Apoptosis-Inducing Ligand (TRAIL), interferon-γ-induced protein-10 (IP-10), and CRP to generate a bacterial versus viral likelihood score. MMBV builds on the performance of CRP by including two additional biomarkers to improve diagnostic accuracy. MMBV has demonstrated high diagnostic accuracy in pediatric febrile patients, outperforming conventional markers when evaluated against expert adjudication [22,23,24]. In the OPPORTUNITY study, MMBV achieved 86.7% sensitivity and 91.1% specificity in differentiating bacterial from viral infections in children with lower respiratory tract infections or fever without source [25]. Early real-world evidence suggests MMBV can influence prescribing behaviors and support appropriate antibiotic use, though further studies are needed to assess its utility across diverse settings and its cost-effectiveness for broader implementation [26,27]. In particular, clinical utility studies are lacking in high-AMR countries, such as Greece, where implementation barriers include cost, clinical familiarity, and integration into existing care pathways.

In this study, we evaluated the clinical utility of MMBV in a Greek pediatric population with acute infections at the time of clinical presentation when clinical uncertainty is highest, using a two-phase design. The pilot phase familiarized clinicians with the test and informed them about the prospective cohort phase. Primary outcomes included antibiotic prescription, diagnostic stewardship, hospitalization rates, and length of stay. We also conducted a cost-impact analysis, populating a published MMBV economic model with local clinical and cost data to assess financial implications within the Greek healthcare system.

## 2. Materials and Methods

### 2.1. Ethics

This study was approved by the Institutional Review Board of University General Hospital ATTIKON, Athens, Greece (IRB reference number: 215/28-03-2023). All data was anonymized. Written informed consent was obtained from all parents or legal guardians of children participating in both the pilot and the prospective cohort study whenever the MMBV score was applied. Procedures complied with the Declaration of Helsinki and relevant national regulations.

### 2.2. Study Design

This single-center, two-phase study was conducted at the Third Department of Pediatrics, University General Hospital ATTIKON, Athens.

During the first phase, the April–June 2023 period, a pilot prospective blinded observational study was conducted. The pilot phase aimed to familiarize clinicians with MMBV and to inform the prospective cohort study. During this stage, patient management by the ED physician was conducted according to standard clinical practice, based solely on clinical presentation and conventional inflammation markers (CBC and CRP). As part of the familiarization process, an adjudication process was conducted (see ‘Clinical Adjudication’).

**Figure 1:**
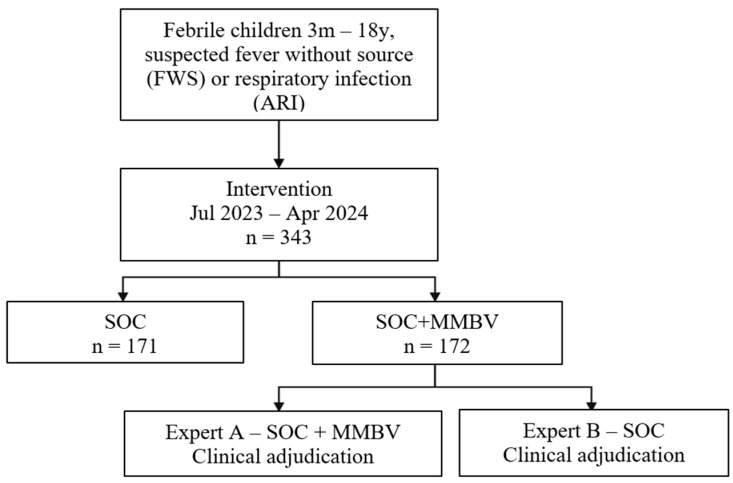
Study flow.

The subsequent prospective cohort phase evaluated the clinical utility of MMBV in pediatric patients with acute infections. Allocation of patients as ‘SOC’ versus ‘SOC+MMBV’ was determined by time of ED presentation. Patients visiting between the hours of 08.00 and 20.00 (daytime) were allocated to SOC+MMBV, while patients visiting between the hours of 20.00 and 08.00 (nighttime) were allocated to SOC (the control group). In the control group, physicians managed patients based on SOC information, whereas in the SOC+MMBV group, physicians had SOC plus MMBV. Of note, physicians had the same probability of being either on the day or the night shift. Thus, this study design mitigated potential physician-related bias yet introduced time-dependent bias (see limitations in the Discussion). To assess the potential impact of MMBV, the two groups were compared in relation to admission rates, antibiotic prescribing (irrespective of administration route), and additional laboratory test ordering. The study flow is detailed in Figure 1.

### 2.3. Eligibility Criteria

Eligible participants were pediatric patients aged 3 months to 18 years with (i) acute fever (≥38 °C) of ≤7 days duration and no other clinical symptoms (fever without source, FWS) or (ii) acute respiratory tract infection (ARI), where ARI symptoms included fever ≤ 7 days and at least one of rhinitis, cough, respiratory distress, ear ache, sore throat—difficulty in swallowing, headache, fatigue/malaise, enlarged lymph nodes, or vomiting/abdominal pain. Eligibility for the SOC+MMBV group included provision of informed consent and a clinical determination by the attending physician that a venous blood draw was warranted. All eligible patients were approached for enrollment. To minimize unnecessary use of the MMBV test and avoid imposing additional venipuncture or excess blood collection on pediatric participants, only patients undergoing routine clinical venous blood testing with remnant blood volume available were included. The typical indications for blood drawing were as follows: a clinically unwell child with dehydration, toxic appearance, or potentially severe disease; duration of fever for more than 4–5 days, with frequent fever spikes; social determinants, such as the family environment of the child. During the entire course of the study, only one parent refused to run the test. At the time of this study, guidelines on antibiotic prescribing in infectious diseases and, specifically, respiratory tract infections by the Hellenic Society for Pediatric Infectious Diseases were not yet published (released in September 2024) [28].

Exclusion criteria aligned with MMBV’s cleared use and included immunosuppression, active chronic inflammatory diseases, chronic viral infection (HIV, HBV, HCV), parasitic or fungal infections, prolonged prior antibiotic exposure (>48 h oral, >12 h IV), and recent major trauma or surgery.

### 2.4. MeMed BV (MMBV)

MMBV (MeMed, Andover, MA, USA) quantifies three host-response biomarkers—TRAIL, IP-10, and CRP—to generate a bacterial vs. viral likelihood score from 0 to 100. Scores below 35 suggest a viral infection, above 65 suggest bacterial, and 35–65 are equivocal. The algorithm is automated [21] and has been clinically validated in multiple studies [22,23,25,29,30,31]. Serum samples were analyzed using the MeMed Key^®^ platform (MeMed, Andover, MA, USA), with results available from serum within approximately 15 min. Although serum samples were used in this study, a whole-blood version is now approved for clinical use.

### 2.5. Clinical Adjudication

In the pilot phase, two Pediatric Infectious Disease (PID) specialists with over 20 years of clinical experience were asked to retrospectively categorize patients as viral, bacterial, or indeterminate and state their intention to order additional tests, administer antibiotic treatment, or admit the patient to the hospital. Initially, they were provided only with the standard of care (SOC) information (medical history and conventional inflammation markers, CBC and CRP). At a later date, expert A (unblinded) was provided with SOC plus MMBV, whereas expert B (blinded) was provided only with SOC.

In the interventional phase, the two PID specialists adjudicated only the SOC+MMBV group. Expert A (unblinded) was provided with SOC information plus MMBV, whereas expert B was provided only with SOC information.

Of note, the experts were not provided with additional laboratory tests (e.g., microbiological findings) that may be ordered as part of routine care. There were no formal guidelines for adjudication, which was based on the data available at presentation.

### 2.6. Study Objectives

The primary objective was to assess the impact of MMBV on physician decision-making in managing pediatric infections. Specifically, the study assesses the following outcomes:

1. Rate of antibiotic prescription (irrespective of administration route), recorded as a categorical variable (yes/no) at any point during the patient’s journey (i.e., ED and/or on the ward during hospitalization). The antibiotic prescription rate reflects antibiotics prescribed and continued after assessment by the ED physicians (e.g., in case a patient is already on antibiotics at presentation). For an antibiotic course to be continued, the attending ED physician would have to consider it necessary.
2. Rate of diagnostic test orders, also assessed as a categorical variable (yes/no), focusing on any diagnostic test used or utilization of urine analysis, urine culture, chest X-ray, blood culture, or multiplex PCR (film-array) testing individually.
3. Hospitalization rate and length of hospital stay (LOS).

To investigate if MMBV availability was associated with optimization of antibiotic prescribing, analyses were stratified into subgroups by hospitalization status or clinical severity, the latter using the NICE ‘fever in under 5 s’ guideline [17]. In addition, analyses were repeated for the sub-cohort of children under 5 years of age, as this is the age group for whom the NICE ‘fever in under 5 s’ risk stratification was validated.

Alignment of antibiotic prescribing with MMBV results (‘MMBV adherence’) was defined as prescribing antibiotics to patients with scores above 65 and not prescribing to patients with scores below 35.

An exploratory objective assessed the cost impact of MMBV implementation using local clinical and cost data.

### 2.7. Cost-Impact Analysis

A previously published MMBV cost-effectiveness model was populated using outcome data from this study [32]. The original model, developed from a UK National Health Service (NHS) perspective, assesses potential cost savings associated with improved diagnostic accuracy and optimized antimicrobial use [32]. Given that our clinical practice stems from NICE guidelines, including patient risk assessment, the model was considered appropriate for adaptation. Adaptation for this study included the following:

1. Incorporation of Greek institutional and national cost data.
2. Use of clinical outcomes from the study cohort.
3. Application of a conversion rate of 1.20 GBP to EUR, and an EUR to GBP conversion rate of 0.84, to preserve cross-model comparability.

The decision-tree (Greek payer perspective, 1-year horizon, cohort *n* = 1000) mirrored the NHS model by Gregg et al., but all clinical probabilities (Table S1) and resource-use frequencies were replaced with study-specific values. Local cost inputs for diagnostics, therapeutics, and bed-days were extracted from ATTIKON hospital finance schedules and public MoH price lists, while admission and readmission tariffs were sourced from a national cost-of-illness analysis for pediatric CAP [33]; full unit costs, utilization rates, and primary data sources are summarized in Supplementary Table S2.

### 2.8. Statistical Analysis

Clinical and demographic patient characteristics in the SOC and SOC+MMBV arms, as well as stratified subgroups, were summarized using descriptive statistics. Continuous variables were reported as medians and interquartile ranges (IQRs), and group differences were assessed using the Mann–Whitney U test. Categorical variables were presented as counts and percentages, with differences between groups evaluated using the Chi-square test or Fisher’s exact test when appropriate. Statistical significance was set at a *p*-value < 0.05. Inter-rater agreement between the two expert adjudicators was evaluated using Cohen’s kappa coefficient. Differences in adjudication status between the experts in the intention-to-treat analysis were assessed with the McNemar test for paired categorical data. All statistical analyses were performed using Python version 3.9.4 19 using the ‘statsmodels’ statistical package.

### 2.9. Sample Size Calculation

No formal sample size calculation was conducted, as the study was pragmatic/exploratory in nature. Consequently, the study’s objectives, including stratified subgroup analyses by hospitalization status and clinical severity, were not powered to detect predefined effect sizes.

## 3. Results

### 3.1. Patient Population

Between July 2023 and April 2024, we recruited 343 patients into the intervention phase of the study (Figure 1); 171 patients were enrolled in the SOC arm and 172 in the SOC+MMBV arm. Only one patient’s parents refused participation in the study during the entire study period. The study arms were similar in sex distribution (36.3% vs. 45.9% female), age (median 5.1 vs. 5.3 years), time from symptom onset (median 3.0 days), and comorbidity rates (17.0% vs. 20.3%); see Table 1 and Table S3. The study population was predominantly composed of older children, with over 85% older than 12 months. Of the 343 patients, 84.5% (290) presented with ARI and 15.5% (53) with FWS. Among SOC+MMBV patients, the test classified 66.3% as viral, 23.3% as bacterial, and 10.5% as equivocal. Antibiotic prescribing aligned with the MMBV results. Antibiotics were prescribed in 18.4% (21/114) of MMBV viral patients, 33.3% (6/18) of MMBV equivocal patients, and 72.5% (29/40) of MMBV bacterial patients. Clinical severity, assessed using the NICE ‘fever in under 5 s’ tool, was comparable between groups, with around half classified as Green risk and approximately 6% as Red risk.

The pathogens listed in ‘respiratory testing’ represent those detected by rapid S. pyogenes test, throat culture, blood culture, and multiplex PCR panels. Pathogen detection rates were calculated as the number of patients in whom the specific pathogen was detected divided by the number of patients in the relevant arm (either SOC or SOC+MMBV) for whom testing was performed.

There was a significant difference in prior antibiotics (50.9% SOC vs. 11.0% SOC+MMBV; *p* < 0.001) and hospitalization rates (36.3% vs. 47.1%; *p* = 0.049) between the SOC and SOC+MMBV arms. Thus, subsequent subgroup analyses by hospitalization status and clinical severity were performed.

### 3.2. MMBV and Antibiotic Prescription

**Figure 2:**
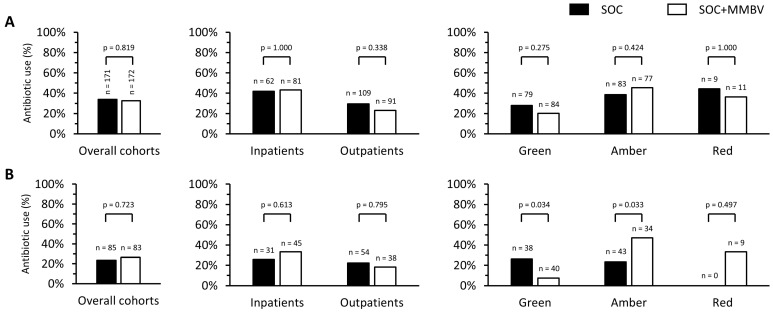
Impact of MeMed BV (MMBV) on antibiotic prescribing. ((**A**)—Upper Panels) Study cohort (3 months–18 years). ((**B**)—Lower Panels) Sub-cohort of patients under 5 years. Black bars represent SOC patients, and white bars represent SOC+MMBV patients. Left panel shows study cohort. Middle panel shows stratification into inpatients versus outpatients. Right panel shows stratification according to the NICE traffic light tool. Green = low risk, Amber = intermediate risk, Red = high risk. Labels above each bar denote number of patients.

Antibiotic prescription rates were assessed across the SOC versus SOC+MMBV groups (Figure 2A) and for the sub-cohort of children under 5 years of age (Figure 2B). There was a pattern of higher prescribing rates in the SOC vs. SOC+MMBV group among outpatients (29.4% (32/109) vs. 23.1% (21/91); *p* = 0.338) and those with a low (‘Green’) risk profile (27.8% (22/79) vs. 20.2% (17/84); *p* = 0.275). When children under 5 years of age with a low risk profile were examined, antibiotic prescription significantly reduced from 26.3% (10/38) in the SOC group to 7.5% (3/40) in the SOC+MMBV group (*p* = 0.034; Figure 2A,B, right panels). Conversely, antibiotic prescription rates increased in inpatients (Figure 2A,B, middle panels) and in those with intermediate (‘Amber’) risk (Figure 2A,B, right panels), corresponding to a higher prevalence of bacterial MMBV scores (35.2%) compared to the Green (13.1%) and Red (18.2%) groups (Figures S1 and S2). The findings were most pronounced in inpatients aged 5 and under. These opposing changes resulted in no net difference in overall antibiotic prescription.

### 3.3. MMBV and Diagnostic Stewardship

**Figure 3:**
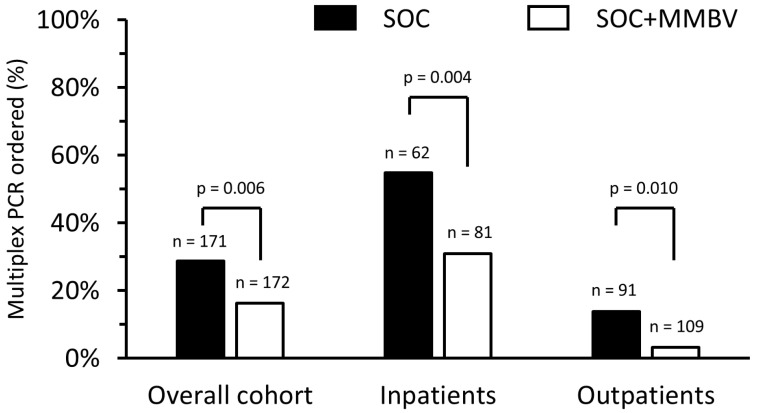
Impact of MeMed BV (MMBV) on respiratory multiplex-PCR utilization. Black bars represent SOC patients, and white bars represent SOC+MMBV patients. Left side shows study cohort, middle shows inpatients, and right side shows outpatients. Labels above each bar denote number of patients.

MMBV had no impact on overall diagnostic test use rates (65.7% (113/172) vs. 68.4% (117/171); *p* = 0.592), nor across subgroups stratified by hospitalization status or clinical severity (Table S4). Specifically, the rate of multiplex PCR panels was significantly lower in the SOC+MMBV arm (28.7% (49/171) vs. 16.3% (28/172); *p* = 0.006), with reduced frequency observed in both inpatients (54.8% (34/62) vs. 30.9% (25/81); *p* = 0.004) and outpatients (13.8% (15/109) vs. 3.9% (3/91); *p* = 0.010) (Figure 3). No differences in order rates in SOC vs. SOC+MMBV were observed for chest X-rays, rapid S. pyogenes antigen tests, throat cultures, urinalysis, or blood cultures (Table S5A).

### 3.4. MMBV and Length of Stay

Mean length of hospital stay did not differ significantly between groups (3.8 ± 3.0 vs. 4.7 ± 3.7 days; *p* = 0.100; Table 1). In the SOC+MMBV arm, admission rates and length of stay increased with clinical severity: admission rates (low = 33.3%; intermediate = 58.4%; high = 72.7%) and LOS (low = 3.2 ± 1.5; intermediate = 5.1 ± 4.1; high = 7.5 ± 4.7 days). No such pattern was observed in the SOC arm.

### 3.5. Clinical Adjudication and MMBV Adherence

In the pilot phase, inter-adjudicator agreement between expert A and expert B (both blinded to MMBV) was 87.5% with moderate concordance based on a Cohen’s kappa value of 0.4 [0.07–0.45]. When unblinded to MMBV results, expert A relabeled 11/48 (22.9%) of patients.

In the intervention phase for the SOC+MMBV arm, the clinical adjudications of expert A (unblinded to MMBV results) for the ARI patients (n = 140) were 22.9% bacterial, 74.3% viral, and 2.1% indeterminate, and one patient was not adjudicated. The clinical adjudications for the FWS patients (n = 32) were 28.1% bacterial, 65.6% viral, and 6.2% indeterminate. These adjudications aligned with the observed antibiotic prescribing in 97.0% of the patients and in 98.8% of the children aged under 5 years. When comparing the clinical adjudications of expert B (blinded to MMBV results) to those of expert A (unblinded to MMBV results), the rate of indeterminate classifications fell from 12.2% to 2.3% (*p* < 0.001) across all ages and from 13.2% to 3.6% (*p* = 0.021) for patients aged 5 years or under.

In the SOC+MMBV arm, physician adherence to MMBV-guided recommendations for antibiotic prescribing was 81.5% across all ages and 82.5% for children aged under 5 years.

### 3.6. Cost-Impact Analysis

We adapted and populated a previously published MMBV cost-effectiveness model using local clinical outcomes and cost data (Tables S1 and S2). In a modeled cohort of 1000 patients reflecting the study population, MMBV implementation was associated with estimated cost savings of EUR 258,156, or EUR 258 per patient. These savings were primarily driven by reduced diagnostic testing (EUR 34,495) and decreased hospital resource utilization (EUR 272,498), offsetting MMBV implementation costs (EUR 51,246). Savings from reduced antibiotic use (EUR 166) and readmissions (EUR 1841) were minimal, indicating the economic benefit was mainly driven by improved diagnostic stewardship and reduced hospitalization costs.

## 4. Discussion

This study adds to the growing real-world evidence that MMBV supports clinical decision-making in pediatric patients with acute infections. Previous interventional studies have demonstrated that MMBV influences prescribing behavior, optimizes antibiotic stewardship, and affects referral patterns from urgent care settings [26,27]. In our cohort, we observed meaningful reductions in antibiotic prescription among outpatients in the emergency department and those with lower clinical severity. This effect was most pronounced in children under 5 years of age with a low-risk clinical profile. Beyond antibiotic prescribing, MMBV also contributed to improved diagnostic stewardship through a significant reduction in multiplex PCR testing. Furthermore, MMBV availability was associated with improved clinical risk assessment, complementing routine patient stratification and management decisions.

Our findings of antibiotic prescribing and resource optimization mirror those reported in recently published real-world evaluations of MMBV in urgent care centers, where MMBV adherence was associated with fewer hospitalizations and contributed to avoiding emergency department referrals [26,27]. Similarly, a pilot randomized controlled trial found that MMBV availability led to optimized antibiotic use and fewer follow-up return visits and hospitalization [34]. Our study adds to the emerging evidence and provides the first real-world pediatric ED insights.

Optimizing antibiotic stewardship through targeted diagnostic interventions is a key pillar of most national AMR action plans [35]. However, stewardship should not rely solely on increased diagnostic testing, as overuse can contribute to resource strain, longer ED wait times, and greater costs—challenges that have intensified since the COVID-19 pandemic [36,37]. Notably, in our study, MMBV supported not only antibiotic stewardship but also diagnostic stewardship by significantly reducing the use of multiplex PCR panels in both admitted in- and outpatients. This is particularly relevant, as many hospital EDs have experienced a sharp increase in multiplex PCR testing since the pandemic, and de-implementation strategies are needed [38,39]. While these tests may enhance infection prevention and control practices, they have shown little impact on antibiotic prescribing or hospital length of stay [19,20]. Moreover, multiplex PCR results often drive increased antiviral prescribing, even in less severely ill patients, despite limited evidence of clinical benefit from antivirals like oseltamivir in mild influenza cases [40]. By integrating host-response information, MMBV may help identify patients who truly benefit from additional pathogen testing. However, it is important to recognize that not all patients require a diagnostic work-up and can be evaluated without any blood drawn. As demonstrated in previous real-world studies, MMBV is most appropriately used in cases where clinical uncertainty persists after initial clinical assessment. Although the pretest probability of a serious bacterial infection is low, concern about missing such patients drives antibiotic overuse. It is precisely in these scenarios that distinguishing between bacterial and viral etiologies is critical.

MMBV was the first advanced host-response assay to obtain regulatory clearance for differentiating bacterial from viral infection. There is one regulatory cleared protein-based test that measures CRP and MxA to assist in distinguishing bacterial from viral infections, but it provides a qualitative result (without any computational algorithm), lending itself to inter-operator variability and low performance [41,42,43]. Recently, a 29-mRNA host-response assay was approved for clinical use only in the United States for adults at the ED [44]. Of note, the multiple studies relating to the development of this tool have employed different biomarkers and/or algorithms, and its sensitivity for detecting bacterial infection is relatively low [45,46,47]. Accordingly, in a recent comparative analysis, this mRNA-based test missed 19% of bacterial infections, whereas MMBV missed only 4% [48]. The present study’s findings further support the utility of MMBV as a rule-out tool for bacterial infection and underscore the need to evaluate each host-response test in the context of its intended use.

Our study has several strengths. It is among the first real-world evaluations of MMBV in a pediatric emergency department setting, conducted in a high-AMR environment. The dataset allowed for detailed stratification, revealing significant reductions in antibiotic use among low-risk (‘Green’) outpatients under 5 years old—a population prone to antibiotic overuse. Another strength of our study is that cases in the SOC+MMBV arm were adjudicated by two PID specialists. The adjudication process, combined with the expert’s intention-to-treat analysis, provided a deeper evaluation of antibiotic prescribing appropriateness, highlighting the potential benefits of incorporating MMBV into clinical decision-making.

On the other hand, our study has notable limitations. The major limitation is the non-randomized, time-dependent design, which introduced a selection bias that likely explains the significantly fewer prior antibiotics and higher admission rate observed for the SOC+MMBV patients, who were recruited exclusively during the day shift. During the daytime, it is less likely that a child would have already been examined and received antibiotics before arriving at the ED. Due to staffing limitations, children are less often hospitalized at night, and patients mostly present because of parental anxiety. We mitigated this time-dependent selection bias partially through subgroup analyses. The bias introduced by the time-dependent design is also manifested by the observation that, among SOC patients, ED physicians often discontinued prior antibiotic therapy after clinical evaluation and diagnosed a viral infection. Conversely, among the SOC+MMBV patients, ED physicians often initiated antibiotic therapy, as children were correctly identified as having bacterial infections, supporting an optimization of antimicrobial stewardship. Notably, the percentage of SOC+MMBV cases that received antibiotics represents the sum of all cases classified as bacterial by MMBV, as well as some of those classified as equivocal.

A second limitation is that social determinants of health, including socioeconomic status and parental factors, may have influenced clinical decisions. In addition, unmeasured confounders, such as seasonal variation, could change the type of pathogens detected in patients, although our study ran throughout a year. Third, MMBV was only applied in the intervention arm, preventing paired comparisons using MMBV score stratification. Fourth, the NICE ‘fever in under 5 s’ traffic light assessment was applied to children of all ages, although the tool is not validated above the age of 5. This was accounted for by stratified analyses only looking at children under the age of 5, where we observed the most significant impact. Fifth, the study was conducted at a single center, and subgroup analyses were underpowered, particularly for high-risk (‘Red’) patients. Sixth, the use of serum-based MMBV testing resulted in longer turnaround times compared to newer whole-blood formats better suited for rapid ED use. Seventh, because our goal was to evaluate the impact of MMBV at the time of presentation, when clinical uncertainty is greatest, we did not analyze discharge diagnoses. Finally, the economic analysis relied on model adaptation rather than prospectively collected cost data, limiting precision in assessing real-world financial impact.

## 5. Conclusions

In conclusion, our study found that MMBV is a valuable adjunctive tool for distinguishing between bacterial and viral infections, supporting improved diagnostic and antibiotic stewardship, particularly in key pediatric subgroups. Even though MMBV comes at a relatively substantial cost for the Greek public healthcare system. If applied in the correct setting using specific guidelines tailored to the areas where its use has resulted in the highest benefit, it can be highly beneficial both for patient management and reducing the cost associated with unnecessary and excessive laboratory workup requested in cases that pose a diagnostic challenge. These arguments would make its implementation more feasible in our public healthcare system.

## Figures and Tables

**Table 1:** Patient demographics and clinical characteristics.

	SOC (*n* = 171)	SOC+MMBV (*n* = 172)
Sex, f; *n* (%)	62 (36.3%)	79 (45.9%)
Age, y; median (IQR)	5.1 (7.3)	5.3 (7.7)
<3 m	0 (0%)	1 (0.6%)
3 m–12 m	17 (9.9%)	23 (13.4%)
>12 m	154 (90.1%)	148 (86.0%)
Time from symptom onset, median (IQR)	3.0 (3.0)	3.0 (4.0)
Temperature, °C; median (IQR)	39.3 (0.9)	39.1 (1.2)
Comorbidities present *	29 (17.0%)	35 (20.3%)
Antibiotics in the last 48 h	87 (50.9%)	19 (11.0%)
Clinical syndrome (at presentation)		
Acute respiratory tract infection (ARI)	150 (87.7%)	140 (81.4%)
Fever without source (FWS)	21 (12.3%)	32 (18.6%)
NICE: Fever under 5 stratification		
Green	79 (46.2%)	84 (48.8%)
Amber	83 (48.5%)	77 (44.8%)
Red	9 (5.3%)	11 (6.4%)
Blood work		
CRP, mg/L; median (IQR)	27.4 (50.6)	25.2 (74.4)
WBC, ×10^9^/L; median (IQR)	11.5 (8.2)	12.6 (8.8)
MMBV score; median (IQR)	N.A.	15.0 (49.0)
MMBV; *n* (%)		
Viral	N.A.	114 (66.3%)
Bacterial	N.A.	40 (23.3%)
Equivocal	N.A.	18 (10.5%)
Respiratory testing; *n* (%)		
Adenovirus	9 (18.4%)	6 (21.4%)
Human rhino/enterovirus	16 (32.7%)	9 (32.1%)
SARS-CoV-2	4 (3.2%)	9 (6.7%)
S. pyogenes	13 (29.5%)	13 (28.3%)
Coronavirus NL63	1 (2.0%)	0 (0.0%)
Influenza	21 (16.9%)	25 (18.7%)

IQR, interquartile range; CRP, C-reactive protein; WBC, white blood count; MMBV, MeMed BV. * Table S3: Patient comorbidity data.

## Data Availability

The data can be made available upon reasonable request.

## Supplementary Materials

The following supporting information can be downloaded at https://www.mdpi.com/article/10.3390/children12091129/s1. Table S1: Base case clinical decision inputs. Table S2: Base case cost and resource use inputs. Table S3: Patient comorbidity data. Table S4: Secondary outcomes for the full cohort and stratified by hospitalization status and clinical severity. Table S5: MMBV impact on use of additional diagnostics. Figure S1: Distribution of MMBV scores indicative of a bacterial immune response across clinical subgroups. Figure S2: Proportion of bacterial MMBV scores stratified by antibiotic prescribing and clinical severity.

## Abbreviations

The following abbreviations are used in this manuscript:

AMR	Antimicrobial Resistance
ARI	Acute Respiratory Tract Infections
CRP	C-reactive Protein
ED	Emergency Department
EEA	European Economic Area
EU	European Union
HBV	Hepatitis B Virus
HCV	Hepatitis C Virus
HIV	Human Immunodeficiency Virus
IP-10	Interferon-Gamma-Induced Protein 10
IQR	Interquartile Range
IV	Intravenous
LOS	Length of Stay
MDR	Multidrug Resistance
MMBV	MeMed BV
NHS	National Health Service
NICE	National Institute for Care Excellence
PCR	Polymerase Chain Reaction
PCT	Procalcitonin
PID	Pediatric Infectious Diseases
SOC	Standard of Care
TRAIL	Tumor Necrosis Factor-Related Apoptosis-Inducing Ligand
UK	United Kingdom
WBC	White Blood Count
