# Supplementary material for: Real-World Utility of the Host-Response MeMed BV Test in a Pediatric Emergency Department: A Non-Randomized Study with Optimized Antimicrobial and Diagnostic Stewardship
Source: Children (Basel). 2025 Aug 27;12(9):1129. doi: 10.3390/children12091129 (PMC12469186; doi:10.3390/children12091129)
Supplement: Supplementary file 1 [file children-12-01129-s001.zip › children-3782590-supplementary.pdf]

Real-World Utility Of The Host-Response MeMed BV test In A Pediatric Emergency Department: Optimized Antimicrobial And Diagnostic Stewardship.

Diamantopoulou P. et al. – Supplemental data file

Table S1: Patient comorbidity data.

	Intervention (n = 343)
Prematurity	7 (2.0%)
Asthma	6 (1.7%)
Autism Spectrum Disorder (ASD)	3 (0.9%)
Cerebral Palsy	3 (0.9%)
Hypothyroidism	3 (0.9%)
Allergic Rhinitis	2 (0.6%)
Developmental Delay	1 (0.3%)
Anemia of Infancy	1 (0.3%)
Eczema	1 (0.3%)
Unilateral Renal Agenesis	1 (0.3%)
Atopic Dermatitis	1 (0.3%)
Propionic Acidemia	1 (0.3%)
Depression	1 (0.3%)
Atypical Systemic Lupus Erythematosus (SLE)	1 (0.3%)
Pulmonary Valve Stenosis (Post-Surgical) with Pulmonary Regurgitation (3rd Degree)	1 (0.3%)
Proteinuria and Arterial Hypertension; Congenital Cataract (Post-Surgical Repair)	1 (0.3%)
Arthritis (Left Knee)	1 (0.3%)
Spastic Tetraplegia	1 (0.3%)
Wolf-Hirschhorn Syndrome	1 (0.3%)
Hydrocephalus	1 (0.3%)
Attention-Deficit/Hyperactivity Disorder (ADHD)	1 (0.3%)
Chronic Obstructive Pulmonary Disease (COPD)	1 (0.3%)
Viral Wheezing	1 (0.3%)
Neurologic Condition	1 (0.3%)
Lymphedema	1 (0.3%)
Noonan Syndrome-like	1 (0.3%)
Chronic Bronchitis	1 (0.3%)
Overgrowth Syndrome	1 (0.3%)
Anemia with Splenomegaly	1 (0.3%)

Table S2: Secondary outcomes for the full cohort and stratified by hospitalization status and clinical severity.

	Control	Cases	p-value
**Overall cohort**			
Admission rate; (%)	36.3%	47.1%	0.049
Length of stay (LOS), dd ; mean (SD)	3.8 (3.0)	4.7 (3.7)	0.100
Additional diagnostics ordered; n (%)	68.4%	65.7%	0.646
Stratification by admission status			
Additional diagnostics ordered; n (%)			
Inpatients	87.1%	75.3%	0.092
Outpatients	57.8%	57.1%	1.000
**Clinical severity (stratified by 'Fever in under 5s' guideline - traffic light assessment**			
Low ('Green')			
Admission rate; (%)	30.4%	33.3%	0.738
Length of stay (LOS), dd ; mean (SD)	3.9 (4.3)	3.2 (1.5)	0.479
Additional diagnostics ordered; (%)	72.2%	59.2%	0.101
Intermediate ('Amber')			
Admission rate; (%)	34.9%	58.4%	0.004
Length of stay (LOS), dd ; mean (SD)	3.8 (2.1)	5.1 (4.1)	0.120
Additional diagnostics ordered; n (%)	62.7%	71.4%	0.246
High ('Red')			
Admission rate; (%)	100.0%	72.7%	0.218
Length of stay (LOS), dd ; mean (SD)	3.2 (1.3)	7.5 (4.7)	0.019
Additional diagnostics ordered; n (%)	88.9%	72.7%	0.591

Table S3. MMBV impact on use of additional diagnostics.

	Control	Cases	p-value
**Overall cohort; n (%)**			
Chest x-ray	23 (13.5%)	24 (14.0%)	0.892
Rapid S. pyogenes test	42 (24.6%)	42 (24.4%)	0.976
Throat swab – culture	14 (8.2%)	16 (9.3%)	0.715
Urinalysis	33 (19.3%)	36 (20.9%)	0.707
Blood culture	34 (19.9%)	31 (18.0%)	0.661
Multiplex PCR panels	49 (28.7%)	28 (16.3%)	0.006
**Inpatient cohort; n (%)**			
Chest x-ray	12 (19.4%)	12 (14.8%)	0.473
Rapid S. pyogenes test	15 (24.2%)	16 (19.8%)	0.525
Throat swab – culture	6 (9.7%)	4 (4.9%)	0.272
Urinalysis	20 (32.3%)	26 (32.1%)	0.984
Blood culture	28 (45.2%)	29 (35.8%)	0.259
Multiplex PCR panels	34 (54.8%)	25 (30.9%)	0.004
**Outpatient cohort; n (%)**			
Chest x-ray	11 (10.1%)	12 (13.2%)	0.496
Rapid S. pyogenes test	27 (24.8%)	26 (28.6%)	0.545
Throat swab – culture	8 (7.3%)	12 (13.2%)	0.171
Urinalysis	13 (11.9%)	10 (11.0%)	0.836
Blood culture	6 (5.5%)	2 (2.2%)	0.236
Multiplex PCR panels	15 (13.8%)	3 (3.3%)	0.010

Table S4. Base case clinical decision inputs

		SOC	SOC+MMBV	Reference
**I. Infectious etiology**				
Children	% Bacterial	24.7%		Study data
	% Viral	75.3%		
**II. Proportion of patients with diagnostic work-up**				
% Test ordered		65.7%		Study data
% No tests ordered		34.3%		
**III. MMBV score distribution**				
Children	% Bacterial	/	23.3%	Study data
	% Viral	/	66.3%	
	% Equivocal	/	10.5%	
**IV. Diagnostic accuracy estimates**				
Children	Sensitivity	91.35%	93.27%	1
	Specificity	70.38%	90.92%	
**V. Detection rates**				
Children	True positives	22.60%	22.90%	/
	False negatives	2.10%	1.80%	
	False positives	22.3°%	12.10%	
	True negatives	53.00%	63.20%	
**VI. Co-infection scenario**				
Children	Viral PCR positive, bacterial outcome	8.90%		1
	Bacterial-viral co-infection detection rate	89.40%		

Abbreviations: MMBV – MeMed BV; SOC – standard of care.

Table S5. Base case cost and resource use inputs

I. Diagnostic test costs and resource use

Parameter	Unit cost	Source	Resource use (Yes = 1 ; No = 0 : [% patients]) Children	Source
Throat swab – culture	€5.22	Local cost data	1 [8.2%]	Study data
Chest X-ray	€4.05	Local cost data	1 [13.5%]	Study data
Blood culture	€5.22	Local cost data	1 [19.9%]	Study data
Multiplex PCR panel	€190	Local cost data	1 [28.7%]	Study data
Urinalysis	€1.76	Local cost data	1 [19.3%]	Study data
Rapid Strep A test	€5.35	Local cost data	1 [24.6%]	Study data
MMBV	€77.74	1	1 [100%]	[1]

II. Antibiotic decision making

IIa. Treatment unit cost

Parameter	Mg / capsule	Pack size	Price / pack	Numbers of capsules per day		Source
				Adults	Children	
Amoxicillin	500mg	21 tables	€0.25	3 tablets	3 tablets	1

IIb Treatment length (per episode)

Parameter	Number of days		% Patients		Source
	Children		Children		
Correct bacterial diagnosis	8.0 days		100%		1
Correct viral diagnosis	0 days		0%		
Diagnosed viral but have bacterial	0 days		0%		
Diagnosed bacterial but have viral	12.0 days	12.0 days	100%	100%	

III. Hospital admission due to misdiagnosis

Parameter	Children		Source
Diagnosed viral but have a bacterial infection			
Proportion of patients who require admission	36.3%		Study data
Total cost per admission	€7034.01		2
Diagnosed bacterial but have a viral infection			
Time spent as an inpatient	84.0 hours		Study data
Cost per excess bed day	€619.7		2
Cost per hour	£25.82		/

IV. Hospital re-admission due to misdiagnosis

Parameter	Adults	Children		Source
Proportion of patients who require readmission	10%	10%		1
Total cost per re-admission	€7034.01			35

Abbreviations: MMBV – MeMed BV.

1. Gregg E, Graziadio S, Green W, et al. Host-response testing with MeMed BV in community-acquired pneumonia: an economic evaluation from the UK NHS perspective. JAC-Antimicrob Resist. 2025;7(1):dlaf016.

2. Naoum P, Athanasakis K, Kyriopoulos I, Liapikou A, Toumbis M, Kyriopoulos J. Community acquired pneumonia: a cost-of-illness analysis in Greece. Rural Remote Health. 2020.

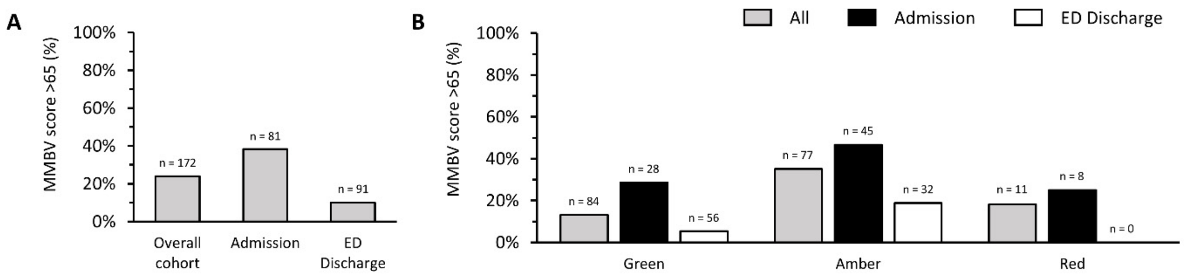

Figure S1: Distribution of MMBV scores indicative of a bacterial immune response across clinical subgroups. (A) Proportion of patients with MMBV scores suggestive of a bacterial infection in the overall study cohort, among in- and outpatients. (B) Proportion of bacterial MMBV scores stratified by clinical severity according to the NICE traffic light system and hospital admission status.

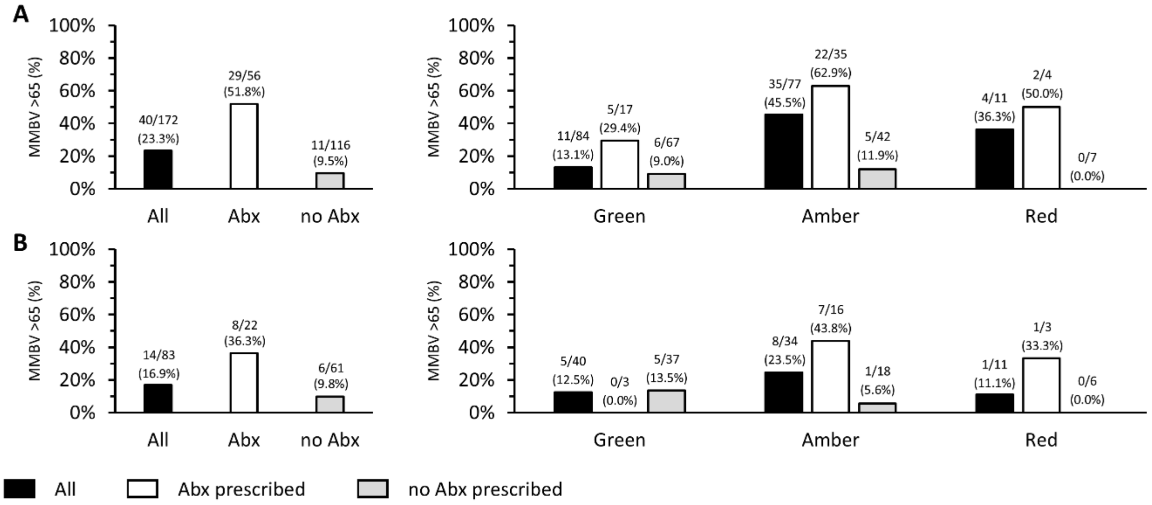

Figure S2: Proportion of bacterial MMBV scores stratified by antibiotic prescribing and clinical severity. Panels A presents data for the overall pediatric cohort, while panels B focus on children under 5 years of age. Plotted, from left to right, are proportion of patients with a MMBV score indicative of a bacterial etiology (>65) as function of antibiotic prescribing practice (left) and 'Fever under 5' traffic light risk assessment (right). Data is plotted for all cases (black) and those that were prescribed (white) or not prescribed (grey) antibiotics.
